# A novel droplet digital PCR human mtDNA assay for fecal source tracking

**DOI:** 10.1016/j.watres.2020.116085

**Published:** 2020-09-15

**Authors:** Kevin Zhu, Brittany Suttner, Amy Pickering, Konstantinos T. Konstantinidis, Joe Brown

**Affiliations:** a School of Civil and Environmental Engineering, Georgia Institute of Technology, Atlanta, GA, USA; b Civil and Environmental Engineering, Tufts University, Medford, MA, USA

**Keywords:** Fecal source tracking, Human mtDNA, Digital droplet PCR, Analytical limit of detection, Analytical lower limit of quantification

## Abstract

Human mitochondrial DNA provides a promising target for fecal source tracking because it is unique and intrinsic to humans. We developed a TaqMan chemistry assay, hCYTB484, targeting the cytochrome *b* gene of the human mitochondrial genome on a droplet digital PCR (ddPCR) platform and compared the performance of hCYTB484 with the HF183/BacR287 assay, a widely used assay targeting human-associated *Bacteroides*. For both assays, we defined the analytical limit of detection and analytical lower limit of quantification using frequency of detection and imprecision goals, respectively. We then established these analytical limits using empirical ddPCR data, presenting a novel approach to determining the analytical lower limit of quantification. We evaluated assay sensitivity using individual human feces from US, Bangladesh, and Mozambique and evaluated assay specificity using cow, pig, chicken, and goat samples collected from the US. To compare assay performance across a range of thresholds, we utilized receiver operating characteristic curves. The hCYTB484 marker was detected and quantifiable in 100% of the human feces from the 3 geographical distant regions whereas the HF183/BacR287 marker was detectable and quantifiable in 51% and 31% (respectively) of human feces samples. The hCYTB484 marker also was more specific (97%), having fewer detections in pig, chicken, and goat samples than the HF183/BacR287 marker (80%). The higher performance of the hCYTB484 marker in individual feces from geographically distant regions is desirable in the detection of fecal pollution from sources to which fewer individuals contribute, such as the non-sewered forms of sanitation (e.g. pit latrines and septic tanks) that serve most of Earth’s population and carry the highest risk of exposure to fecal-oral pathogens.

## Highlights

• We developed a novel TaqMan chemistry assay for the ddPCR platform targeting human mtDNA.
• mtDNA was detected and quantifiable in 100% of human feces samples from the US, Mozambique, and Bangladesh.
• mtDNA can complement fecal source tracking methods.

## Graphical abstract

**Figure:**
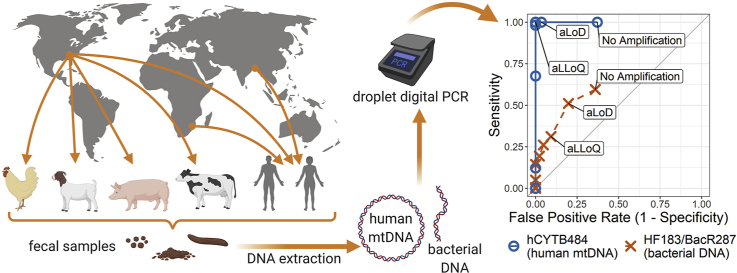

## 1 Introduction

Within the fecal source tracking (FST) field, the development of a human-specific, culture-, and library-independent marker has been a goal for many research efforts due to its potential to be an ideal, rapid, and broadly-applicable method (Boehm et al., 2013; Green et al., 2014; Stachler et al., 2017; U.S. Environmental Protection Agency (EPA), 2019). The challenge that remains is the development of such a marker that also retains high sensitivity and specificity across geographies. As classification methods, FST markers are frequently evaluated on sensitivity and specificity performances. Sensitivity is calculated by the number of true positives divided by the sum of true positives and false negatives. Specificity is calculated by the number of true negatives divided by the sum of true negatives and false positives. Studies have demonstrated variable specificity for human-associated FST markers targeting microbial DNA across varying geographies (Boehm et al., 2013; Harris et al., 2016; Nshimyimana et al., 2017; Odagiri et al., 2015; Reischer et al., 2013), possibly influenced by variations in the human gut microbiome (Yatsunenko et al., 2012) and human-animal interactions (Harris et al., 2016; Nshimyimana et al., 2017). Microbial DNA markers have also exhibited variable sensitivity across different scales of fecal pollution around the globe: high sensitivity in wastewater collected from sewage networks but lower sensitivity in human fecal samples (Mayer et al., 2018). While much effort has been made towards the development of human-specific, culture-, and library-independent markers, the variable performance of such microbial markers across various settings necessitates their local validation in the intended setting prior to use to ensure that the fecal signal can be unambiguously attributed to a source (Harris et al., 2016; Nshimyimana et al., 2017; Odagiri et al., 2015).

Most of the global human population does not have access to sewered sanitation: an estimated 56% of households globally have unsewered sanitation facilities and 12% of households globally have no sanitation facility (Berendes et al., 2018). Furthermore, disproportionalities in sanitation exist. The same geographic regions that contain the lowest rates of sewered households are where most of the human population live and where the most human feces are produced (Berendes et al., 2018). This means that for much of the world’s population, enteric pathogen exposure risks are related to sludges and other non-sewered fecal waste streams that are composites of feces from relatively few individuals. This reality underscores the need for markers to serve in situations where exposure to fecal sludge is more relevant than exposure to wastewater collected through a sewerage network. In these settings, markers that are widely distributed across humans are desired because with fewer individuals contributing to fecal sludges the presence of the marker in each individual is crucial.

Because mtDNA methods directly target conserved host DNA sequences present in individuals across the species instead of host-associated markers, the link between host and fecal indicator microbe is not relevant, and, therefore, mtDNA methods may not require the same extent of validation as emphasized for host-associated, microbial DNA markers. Additionally, because mtDNA is universally distributed across human individuals, mtDNA methods may offer superior sensitivity in individual feces and in scenarios where relatively fewer individuals contribute to the fecal pollution. Previously-published quantitative PCR (qPCR) human mtDNA-based assays for FST have exhibited high specificity in the US (Caldwell et al., 2007; Schill and Mathes, 2008) and in China (He et al., 2016); however, concentrations of mtDNA-based markers are typically found in lower numbers in sewage samples and in anthropogenically impacted water environments relative to microbial-based FST markers (He et al., 2016; Schill and Mathes, 2008; Stea et al., 2015; Villemur et al., 2015). Because mtDNA-based markers are typically found at lower concentrations than microbial-based markers, we utilized the droplet digital PCR (ddPCR) platform for its improved reproducibility in lower concentrations. As a tool for FST, ddPCR has demonstrated several appealing characteristics when compared to qPCR, including greater tolerance of PCR inhibitors (Cao et al., 2015), reduced quantification bias when duplexing assays (Cao et al., 2015) and improved reproducibility over qPCR (Hindson et al., 2013; Strain et al., 2013).

In this study, we designed a novel assay, hCYTB484, targeting human mtDNA on the ddPCR platform with three design goals: 1) specificity to human mtDNA, 2) optimization for ddPCR conditions, and 3) avoidance of identified polymorphisms for a geographical stable assay. We then characterized the analytical performance of hCYTB484 alongside another FST assay, the HF183/BacR287 assay, a high performing assay targeting human-associated *Bacteroides*, in parallel on ddPCR. The HF183/BacR287 assay (Green et al., 2014) is an improved version of a widely-studied microbial DNA assay (Haugland et al., 2010). For both assays, we defined detection and quantification goals based on frequency of detection and imprecision, respectively. Using these goals, we established the limit above which samples are considered positive for the target, referred to as the analytical limit of detection (aLoD) and the limit above which samples are considered quantifiable for the target, referred to as the analytical lower limit of quantification (aLLoQ). To investigate the geographic variability of the two assays in situations where few individuals contribute to the fecal pollution, we tested the assays on human feces obtained from the US, Bangladesh, and Mozambique. We then compared sensitivity and specificity performances between the two assays in fecal samples by using receiver operating characteristic (ROC) curves to detail assay performance beyond scalar values of sensitivity and specificity. We hypothesized that by targeting a widely conserved host-mtDNA marker, we would develop an assay that is more sensitive in human feces from various geographies than the microbial-based HF183/BacR287 assay.

## 2 Materials and methods

### 2.1 Assay design

We designed our assay within the cytochrome *b* gene because this region has been used to differentiate between species even in short fragments, 358 (Parson et al., 2000) and 148 bp (Lopez-Oceja et al., 2016). We downloaded complete cytochrome *b* sequences from GenBank (Clark et al., 2016) (see supplementary materials for accession numbers) and produced a consensus sequence for human, pig, cow, goat, chicken, dog, rat, and white-tailed deer using Jalview (version 2.10.3). From the consensus sequences, we performed an alignment of the consensus sequences using Mafft (L–INS–i algorithm, version 7.394). We selected a region with high differentiation between species guided by the species-variable regions identified in Lopez-Oceja et al. (2016) and then entered that region into the Integrated DNA Technologies, Inc. (IDT, Coralville, IA, US) PrimerQuest online tool along with ddPCR conditions specified by Bio-Rad (primer concentration at 900 nM, probe concentration at 250 nM, concentration of divalent cations at 3.8 mM, and the concentration of dNTPs at 0.8 mM) to design forward primer, reverse primer, and probe sequences.

Once PrimerQuest output a set of designed assays, we searched the IDT-designed primer and probe sequences against human and non-human mtDNA sequences downloaded from GenBank to evaluate the *in-silico* specificity of the sequences using the NCBI BLAST tool (Camacho et al., 2009). When searching against all human mtDNA sequences downloaded from NCBI at the time (March 2018), we looked for oligonucleotide sequences that had the most returns with 100% identity and full coverage across the oligonucleotide sequence. When searching against downloaded non-human mtDNA sequences, we looked for primer sequences that had the fewest returns with the lowest percent identity and emphasized a lack of identity at the 3’ end of the primers to maximize specificity to humans (Wright et al., 2014). Because mismatches between template and oligonucleotides can result in inefficiencies in PCR (Letowski et al., 2004; Stadhouders et al., 2010), we identified two frequently described polymorphisms within the region we were designing in: the 15301 and 15326 positions (Ablimit et al., 2013; Hwa et al., 2010; Lee et al., 2002), relative to the revised Cambridge Reference Sequence, rCRS (Bandelt et al., 2014) and avoided these two polymorphisms during the primer and probe design process.

### 2.2 Samples

To evaluate the sensitivity of the assay, we used both human fecal samples and human wastewater samples. We obtained feces samples from the US, Bangladesh, and Mozambique and obtained municipal wastewater samples from the Atlanta, GA, US metropolitan region, including 6 primary influent samples and 1 primary effluent sample from plants ranging in treatment capacity from 16 to 90 million gallons per day (73–410 million liters per day). To test specificity against non-human sources, we collected fecal samples from 22 cows, 35 pigs, and 22 goats as well as 8 samples of chicken litter from farms located in north Georgia. To test the environmental application of hCYTB484, we collected freshwater samples from the Chattahoochee River (Atlanta, GA, US) at 5 different sampling locations on 3 different days starting approximately 5 km downstream of Buford Dam and continuing downstream in approximately 25 km increments through the Atlanta metropolitan area (see Fig. S4 for a map of sampling locations). Using vacuum filtration, we filtered each wastewater and freshwater sample in duplicate, with volumes ranging from 25 to 50 mL for wastewater and 500–750 mL for freshwater onto sterilized 0.22 μm pore size, 47 mm diameter, asymmetrical polyethersulfone membrane filters (MilliporeSigma, Burlington, MA, US). We then stored all filters and samples at −80 °C until nucleic acid extraction. See supplementary materials for more details.

### 2.3 Nucleic acid extractions

We extracted the human feces samples from the US using the Qiagen (Hilden, Germany) PowerSoil kit according to the manufacturer’s protocol and stored eluted DNA in Buffer C6 at −20 °C until analysis. We extracted human feces samples from Mozambique and Bangladesh as well as the chicken litter samples using the Qiagen QIAamp 96 PowerFecal QIAcube HT kit on the QIAcube HT platform and stored eluted DNA in Buffer ATE at −80 °C in low-retention microcentrifuge tubes until analysis. We extracted cow, pig, and goat feces samples using the Qiagen PowerSoil kit according to the Human Gut Microbiome Project protocol and stored eluted DNA in Buffer C6 at −80 °C in low-retention microcentrifuge tubes until analysis. We extracted DNA from freshwater and wastewater sample filters using the Qiagen PowerWater kit according to the manufacturer’s protocol and stored eluted DNA in Buffer EB at −80 °C in low-retention microcentrifuge tubes until analysis. We assayed all extracts with the Qubit™ dsDNA HS assay (Invitrogen™, Carlsbad, CA, US) to quantify dsDNA concentration. We performed extraction blanks with each set of extraction and tested each extraction blank with the Qubit dsDNA HS, hCYTB484, and HF183/BacR287 assays.

### 2.4 ddPCR

#### 2.4.1 ddPCR experimental conditions

In reporting the methods and materials for this study, we referenced the Minimum Information for Publication of Quantitative Digital PCR Experiments Guidelines (Huggett et al., 2013). We performed our ddPCR experiments on a Bio-Rad QX200™ Droplet Digital™ PCR System in our laboratory. For each ddPCR well, we created a 21 μL PCR mixture containing 1x Bio-Rad ddPCR™ Supermix for Probes, primer (Table 1), 250 nM of probe (Table 1), and 2 μL of template. For any sample outside of the dynamic range of the QX200™ system, we made tenfold dilutions until we could obtain a result within the dynamic range. We did not use additives in the reaction mixture or enzymatic digestion on samples. We performed droplet generation using 20 μL of reaction mixture and 70 μL of Bio-Rad Droplet Generation Oil for Probes with the Bio-Rad QX200 Droplet Generator. We then transferred the oil emulsion using a multichannel pipettor to a ddPCR™ 96-Well Plate. After sealing the plate using the Bio-Rad PX1 PCR Plate Sealer with a foil PCR Plate Heat Seal, we loaded each plate onto the Bio-Rad C1000 Touch™ Thermal Cycler. For each thermal cycler routine, we used 10 min at 95 °C, followed by 40 cycles of 30 s at 95 °C and 60 s at the assay-specific annealing temperature (annealing temperature of 58 °C for HF183/BacR287 and 59 °C for hCYTB484), followed by a 10-min hold at 98 °C with a ramp rate of 2 °C/s. We read plates with the Bio-Rad QX200 Droplet Reader set to the absolute quantification experiment setting. We did not measure our partition volumes (Košir et al., 2017) but utilized the assumed partition volume of 0.85 nL in Bio-Rad QuantaSoft (Version 1.7.4.0917).

**Table 1:** Oligonucleotide sequences used in this study.

Oligonucleotide	Sequence (5’ → 3′)	Reference	Notes
hCYTB484F	CAATGAATCTGAGGAGGCTAC	This study	121 bp amplicon
hCYTB604R	CGTGCAAGAATAGGAGGTG	This study	
hCYTB520TM	FAM-ACCCTCACACGATTCTTTACCTTTCACT-BHQ	This study	IDT Zen quencher
HF183	ATCATGAGTTCACATGTCCG	Bernhard and Field (2000)	126 bp amplicon
BacR287	CTTCCTCTCAGAACCCCTATCC	Green et al. (2014)	
BacP234MGB	FAM-CTAATGGAACGCATCCC-MGBNFQ	Green et al. (2014)	Minor Groove Binder
BacP234IAC	HEX-AACACGCCGTTGCTACA-MGBNFQ	Green et al. (2014)	
HF183/BacR287 IAC	ATCATGAGTTCACATGTCCGCATGATTAAAGGTATTTTCCGGTAGACGATGTGTAGCAACGGCGTGTTATAGTAGGCGGGGTAACGGCCCACCTAGTCAACGATGGATAGGGGTTCTGAGAGGAAG	Green et al. (2014)	Used as an IDT gBlocks®
HF183/BacR287 Plasmid	ATCATGAGTTCACATGTCCGCATGATTAAAGGTATTTTCCGGTAGACGATGGGGATGCGTTCCATTAGATAGTAGGCGGGGTAACGGCCCACCTAGTCAACGATGGATAGGGGTTCTGAGAGGAAG	Green et al. (2014)	(only amplicon sequence shown)
hCYTB484 positive control (IDT gBlocks®)	CAATGAATCTGAGGAGGCTACTCAGTAGACAGTCCCACCCTCACACGATTCTTTACCTTTCACTTCATCTTACCCTTCATTATTGCAGCCCTAGCAGCACTCCACCTCCTATTCTTGCACG	This study	(only amplicon sequence shown)

#### 2.4.2 ddPCR positive controls

For the HF183/BacR287 assay, we constructed plasmids from positive human feces samples, cloned using an Invitrogen™ (Carlsbad, CA, US) TOPO®TA Cloning® kit with the pCR™4-TOPO vector and verified the plasmid sequences using Sanger sequencing (GENEWIZ, South Plainfield, NJ, US). Prior to use in ddPCR, we digested plasmids using EcoRI (Invitrogen™ Anza Restriction Enzyme). For the hCYTB484 assay, we ordered custom-manufactured, sequence-verified synthesized DNA (gBlock) products manufactured by IDT (Coralville, IA, US). Sequences of oligonucleotides used in this study are shown in Table 1. We used the HF183/BacR287 internal amplification control (IAC) as a gBlock to assess PCR inhibition in each undiluted sample as described (Green et al., 2014). We also monitored each no-template control (UV-treated molecular grade water) and extraction control. If amplification did occur in a no-template control, we re-ran the samples until we obtained a plate with zero positive partitions. If any sample exceeded the ddPCR dynamic range, we reran it with increasing ten-fold dilutions until the well had a ddPCR λ (mean copies per partition) under 6 and was above the aLLoQ.

#### 2.4.3 ddPCR signal

The ddPCR platform discretizes a 20 μL PCR reaction into nanoliter-scale reactions, referred to here as partitions, by emulsifying the PCR reaction within oil droplets. Each ddPCR well contains tens of thousands of such partitions. After thermal cycling the wells, the ddPCR platform reads a presence/absence signal from each partition in a ddPCR well and calculates a concentration from the ratio of the number of positive partitions to the number accepted partitions. The formula (QuantaSoft Version 1.7.4.0917) used to estimate the concentration of target in the original sample can be written as:

$$ddPCR\;concentration\;in\;a\;well=-ln\left(1-\frac{positive\;partitions\;in\;a\;well}{accepted\;partitions\;in\;a\;well}\right)\times \left(\frac{1}{0.85\;nL}\times \frac{1000nL}{1\mu L}\right)$$

From the above equation, the number of positive partitions and accepted partitions represent the raw signal of the ddPCR platform. The ddPCR concentration in a well represents the number of copies of the target found in the 20 μL PCR reaction; we will refer to copies per ddPCR well, which is the ddPCR concentration in a well described above multiplied by 20 μL.

Due to previous experience with partitions of intermediate fluorescence on the ddPCR platform, specifically, in situations where primer and template were mismatched by one or two base pairs, we chose to adopt a moderate approach to distinguishing between positive and negative partitions by setting the threshold midway between the peaks of the negative partitions and the positive partitions to improve discrimination against similar but not exact sequences. We only accepted ddPCR results from wells with more than 10,000 accepted partitions (Fig. S1). Our mean copies per partition within each well was 2.19. We normalized marker counts in feces to ng of dsDNA (as measured by the Qubit HS dsDNA assay, Invitrogen, Carlsbad, CA, US) to account for variability between feces consistencies (Kelty et al., 2012).

### 2.5 Analytical limits

#### 2.5.1 Analytical limit of detection

We assumed that the number of positive partitions per ddPCR well can be modeled using the Poisson distribution (Karlin-Neumann and Bizouarn, 2018). Assuming that the conditions under which each ddPCR well was prepared, thermal cycled, and quantified were equal such that partitions from the same template can be considered to be from the same population, we modeled a Poisson distribution from our observed data using maximum likelihood estimation (MLE). Because the Poisson distribution originates from the exponential family, it can be shown that the MLE is the sample mean.

To assess false-positive rates from known analytical blanks, we assayed UV treated (for 15 min) molecular grade water in 94 replicates with both assays (HF183/BacR287 and hCYTB484). We chose to define the limit of detection as the minimum level of the analyte in a sample that will be reported as detected with 95% probability using a known positive control. We used the Poisson distribution to model the number of positive partitions in a ddPCR well with λ representing the mean number of positive partitions in a well needed to observe *k* positive partitions in a well. To determine the level of analyte (λ) that would be detected 95% of the time in a Poisson process, we set the Poisson probability mass function equal to a probability of 0.05 for observing no positive partitions in a well (*k* = 0).

$$\text{P}\left(k\;\text{positive}\;\text{partitions}\;\text{in}\;\text{a}\;\text{well}\right)=e^{-\lambda }\frac{\lambda^{k}}{k!}$$

$$\text{P}\left(0\;\text{positive}\;\text{partitions}\;\text{in}\;\text{a}\;\text{well}\right)=e^{-\lambda }\frac{\lambda^{0}}{0!}=0.05$$

$$e^{-\lambda }=0.05$$

$$\text{ln}e^{-\lambda }=\text{ln}\left(0.05\right)$$

λ≈3

For both assays, we serial diluted positive controls to approximately 3 positive partitions per well and then assayed the dilution for 46 ddPCR replicates.

#### 2.5.2 Analytical lower limit of quantification

We chose to define the analytical lower limit of quantification as the minimum level of analyte in a sample that will result in a coefficient of variation (CV) of 25% using a known positive control. The target CV of 25% or less is a commonly used aLLoQ goal for ddPCR platforms (Bogožalec Košir et al., 2019; Milosevic et al., 2018). The CV, also referred to as the relative standard deviation (RSD), is defined as the ratio of the standard deviation, σ, to the mean, μ.

$$\text{CV}=\frac{\sigma }{\mu }$$

Because repeated assaying of various target concentrations may be required to meet a specific CV target, we sought to understand how the assay imprecision changes with level of concentration to allow us to interpolate estimates of the level of concentrations needed to meet our target CV. We assayed a variety of concentration levels, calculated CV values, and fitted linear regressions to log_10_-transformed values of CV and copies per ddPCR well (copies per 20 μL well). The regressions fitted for each assay used in this study incorporate some intraassay variability as the 4 estimates of the CV for each assay are from 3 separate serial dilutions created on different days.

### 2.6 Statistical analyses

We completed all statistical analyses, simulations, and graphs in R version 3.6.1.

## 3 Results

### 3.1 Analytical limit of detection

**Fig. 1:**
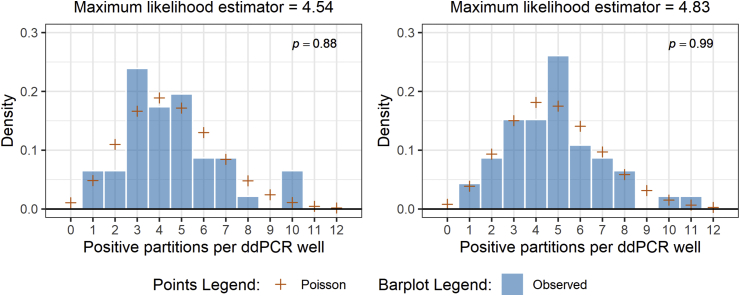
Comparisons of sampling distributions with corresponding expected Poisson distributions generated using maximum likelihood estimation. Left and right plots show results from the hCYTB484 and HF183/BacR287 assays, respectively. P-values from the results of Anderson-Darling tests between theoretical and observed distributions are shown in the top right corners of each plot, indicating that the two distributions are not significantly different.

While approximately 95% of analyzed blanks lacked any amplification for both assays, our results showed that false-positive partitions occurred at low concentrations and low rates for both assays (Table S1). Prior to performing experiments to validate the aLoD, we used the Poisson distribution to calculate an expected value of 3 positive partitions per ddPCR well as the level of concentration within the ddPCR well that would enable us to observe at least 1 positive partition in 95% of ddPCR wells. In our experiments to validate an aLoD of 3 positive partitions per well, we achieved mean positive partitions per well of 4.54 (hCYTB484) and 4.83 (HF183/BacR287). We compared sampling distributions from our ddPCR aLoD experiments with corresponding expected Poisson distributions using the Anderson-Darling test and obtained *p*-values of 0.89 and 0.99 for the hCYTB484 and HF183/BacR287 assays, respectively (Fig. 1).

### 3.2 Analytical lower limit of quantification

**Fig. 2:**
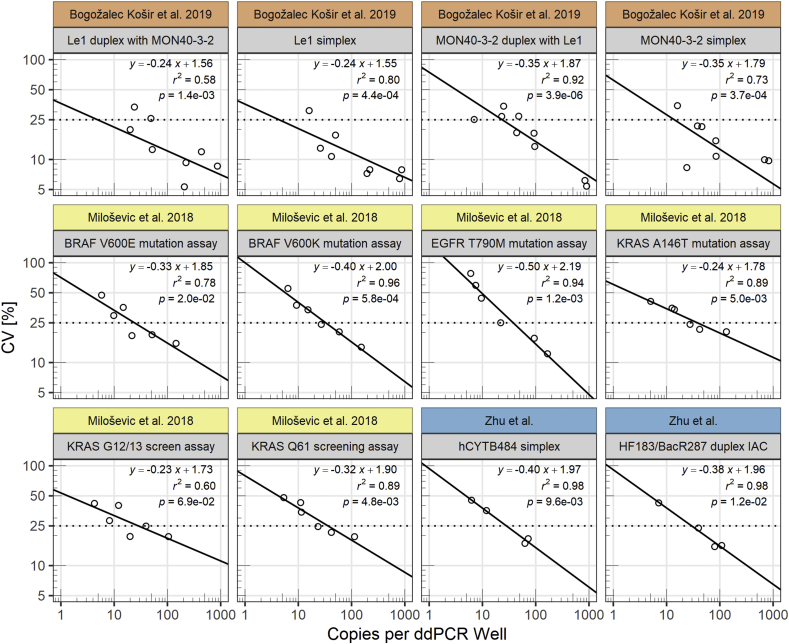
CV values plotted against copies per ddPCR well on log_10_-log_10_ axes for data obtained from this study (Zhu et al.), Bogožalec Košir et al., 2019 (Bogožalec Košir et al., 2019), and Milošević et al., (2018) (Milosevic et al., 2018). We fitted ordinary least squares linear regressions to the log_10_ transformed CV values and log_10_ transformed copies per ddPCR well. We report linear equations, r^2^ values, and F-statistic p-values (for the slope coefficient) displayed in top right corner of each plot and plotted a dotted horizontal line at CV = 25% to represent the aLLoQ imprecision goal. The 12 plots include both simplex and duplex assays with assay name indicated under each study label.

To determine the analytical lower limit of quantification (aLLoQ), we defined our aLLoQ goal as the level of concentration that would result in a coefficient of variation (CV) of the copies per ddPCR well equal to 25%. We interpolated the level of concentration that would allow us to achieve a CV of 25% by fitting a linear regression to log_10_ transformed CV and log_10_ transformed copies per ddPCR well values (Fig. 2). The linear regressions produced aLLoQs of 28.2 and 29.6 copies per ddPCR well for hCYTB484 and HF183/BacR287, respectively. Table 2 summarizes the results of our analytical limit experiments and demonstrates how we classified samples in this study as positive or negative.

**Table 2:** Summary table of the analytical limits established for hCYTB484 and HF183/BacR287 in this study and how the analytical limits were used to determine a sample as positive or not detected.

Threshold Name	Threshold Definition	Sample Status Criteria	Sample Status	Positive vs. Not Detected
No Amplification	0 positive partitions	positive partitions in a ddPCR well = 0	No amplification	Not Detected
		0 < positive partitions in a ddPCR well <3	Amplification below aLoD	Amplification Below Limit of Detection
Analytical Limit of Detection (aLoD)	3 positive partitions			
		3 ≤ positive partitions in a ddPCR well < assay-specific aLLoQ	Detected but not quantifiable	Positive Sample
Analytical Lower Limit of Quantification (aLLoQ)	28.6 copies/ddPCR well (hCYTB484) 29.6 copies/ddPCR well (HF183/BacR287)			
		assay-specific aLLoQ ≤ copies per ddPCR well	Quantifiable	

### 3.3 Sensitivity and specificity results in feces samples

**Fig. 3:**
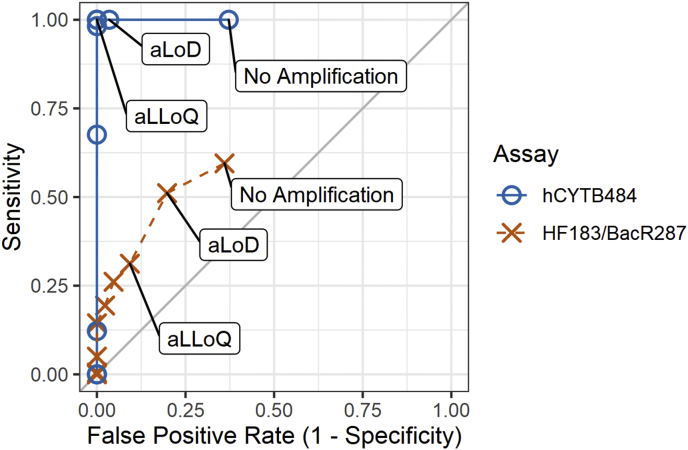
Receiver operating curves for hCYTB484 and HF183/BacR287 ddPCR assays using human feces (n = 222), cow feces (n = 22), pig feces (n = 34), chicken litter (n = 8), and goat feces (n = 22). Rates of sensitivity and false-positives are calculated for a range of thresholds beginning with no amplification, analytical limit of detection (aLoD), and analytical lower limit of quantification (aLLoQ).

We tested the sensitivity and specificity of HF183/BacR287 and hCYTB484 on feces samples from humans, cows, pigs, chickens, and goats (Table 3). For any non-target amplification, we confirmed the presence of the expected size of PCR product on endpoint PCR with gel electrophoresis. We then calculated sensitivity and specificity values for these samples over a range of thresholds (Table 4). By calculating sensitivity and specificity for a range of thresholds, we demonstrate how assay performance can vary based on threshold definition. As the threshold, or the level of concentration at which a well is declared as positive, is increased from aLoD to the aLLoQ, the hCYTB484 assay exhibited an increase in specificity from 97% to 100% while sensitivity remained constant at 100%. Across the same threshold levels, the HF183/BacR287 assay exhibited an increase in specificity from 80% to 92% but with a tradeoff in sensitivity, decreasing from 51% to 31%. We then plotted this dependency as a receiver-operating characteristic (ROC) curve (Fig. 3).

**Table 3:** Background information on the human and non-human fecal samples used in this study and results of specificity and sensitivity (with the threshold at which samples are declared positive or negative set at the aLoD).

	Sample source	No. of samples	Sample type	Source information	No. of samples positive (threshold set at aLoD)	
					**hCYTB484**	**HF183/BacR287**
**Non-human**	Cow	22	Individual	Farm	1 (5%)	0 (0%)
	Pig	34	Individual	Farm	2 (6%)	3 (9%)
	Chicken	8	Litter	Farm	0 (0%)	5 (63%)
	Goat	22	Individual	Farm	0 (0%)	9 (41%)
	Overall Specificity =				97%	80%
**Human**	United States	11	Individual	Adults	11 (100%)	10 (90%)
	Bangladesh	140	Individual	Children	140 (100%)	60 (43%)
	Mozambique	71	Individual	Children	71 (100%)	44 (62%)
	Municipal Wastewater	7	Composite	16 to 90 million gallons per day (73 to 410 million liters per day)	7 (100%)	7 (100%)
	Overall Sensitivity =				100%	51%

**Table 4:** Rates of specificity, sensitivity, and false positives calculated for a range of thresholds for both assays.

Threshold	Units	HF183/BacR287			hCYTB484		
		Specificity	Sensitivity	False-positive Rate	Specificity	Sensitivity	False-positive Rate
No amplification (= 0)	positive partitions per ddPCR well	0.64	0.59	0.36	0.63	1.00	0.37
aLoD (≥3)		0.80	0.51	0.20	0.97	1.00	0.03
aLLoQ (assay dependent)	copies/ddPCR well	0.92	0.31	0.08	1.00	1.00	0.00
10	copies/ng of dsDNA	0.95	0.26	0.05	1.00	1.00	0.00
100		0.98	0.19	0.02	1.00	0.98	0.00
1000		1.00	0.14	0.00	1.00	0.68	0.00
10000		1.00	0.05	0.00	1.00	0.12	0.00
100000		1.00	0.00	0.00	1.00	0.00	0.00

**Fig. 4:**
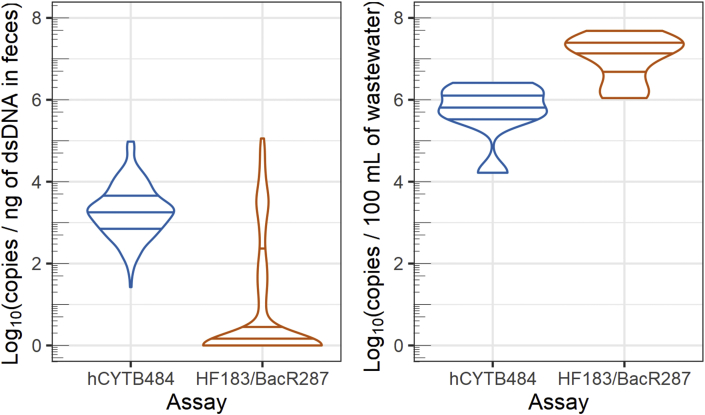
Violin plots of marker concentrations in human feces (left) and human wastewater (right). The violin plots display estimated kernel density along the vertical axis with horizontal lines depicting the 25th, 50th, and 75th quantiles. All concentrations are plotted as log_10_ (concentration + 1) and concentrations from feces are normalized to concentration of dsDNA (ng of dsDNA determined by Qubit).

### 3.4 Wastewater and freshwater samples

**Fig. 5:**
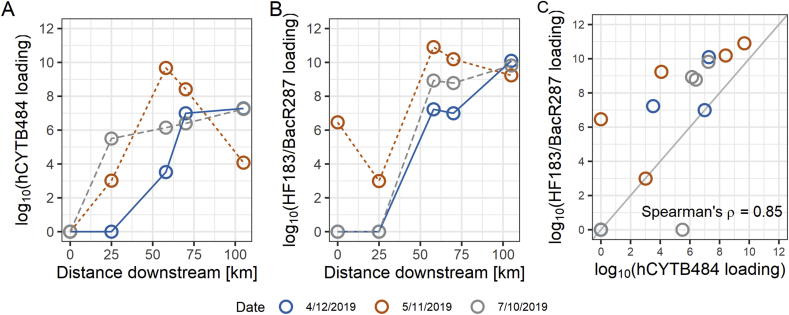
Loadings of gene markers for samples taken along the Chattahoochee River, plotted as copies/mL of sample normalized to discharge as mL/s (data from USGS stream gages at time of collection) to produce copies of marker/second of discharge. The 0 km distance starts approximately 5 km downstream of Buford Dam, and drainage from urban development increases as the relative distance downstream increases. Loading values are plotted as log_10_ (loading + 1). Samples not detected were imputed with a value of zero to allow for visual representation. Samples detected but not quantifiable were imputed to produce a value at the analytical limit of detection to allow for visual representation.

We quantified the hCYTB484 marker at higher levels of concentration than the HF183/BacR287 marker in human feces but found HF183/BacR287 at higher levels of concentration than hCYTB484 in municipal wastewater samples (Fig. 3). In freshwater samples, we were able to quantify both markers at increasing levels of loading (copies per mL normalized to mL per second of discharge) with increasing drainage contribution from urban development (Fig. 5A and B). However, we typically quantified the HF183/BacR287 marker at higher levels than those of the hCYTB484 marker (Fig. 5C).

## 4 Discussion

### 4.1 Analytical limit of detection

It is possible that the hCYTB484 assay exhibited slightly higher rates of false positives because the assay targets human mtDNA; there are several opportunities for human mtDNA to contaminate the reaction mixture due to the series of manipulations required for ddPCR. Combining the results from the analytical blanks with the false-positive rates from non-human feces, we show that very low concentrations (below the aLoD) of false positives occur at low rates when using the hCYTB484 assay, and, consequently, we recommend that the threshold be set at or above the aLoD and care be taken to avoid contamination of human mtDNA. We have found that UV treatment of tubes, reaction wells, and ddPCR droplet cartridges as well as the use of a 10% bleach solution to disinfect gloves, pipettes, and contact surfaces after any potential human contact or contamination have aided in successfully avoiding contamination of human mtDNA during sample processing and analysis.

The sampling distributions of positive partitions per ddPCR well in our aLoD ddPCR experiments suggest that the Poisson distribution may be a representative theoretical model for the number of positive partitions per ddPCR well. We note that it is a theoretical stochastic model and does not account for potential biases introduced by external sources of variability (Karlin-Neumann and Bizouarn, 2018), such as the operator or equipment. Using our findings at the mean positive partitions per well of 4.54 and 4.83, we extrapolated our aLoD down to a mean of 3 positive partitions per well using the Poisson distribution and our detection goal of a theoretical 95% probability. In addition to modeling the probability of a detection for an aLoD, it is important to consider the likelihood of an observation. If a single ddPCR well contained 3 positive partitions, one could calculate the likelihood of this observation for a range of λ values, finding that the most likely λ that would produce a well of 3 positive partitions would be a λ of 3. Recognition of the likelihood of an observation is key because a single ddPCR well of a sample is just one observation drawn from a distribution that exists for the sample. Thus, an aLoD that maximizes the likelihood is informative. This aLoD of 3 has been used in both qPCR (Bustin et al., 2009) and ddPCR applications (Cao et al., 2015).

### 4.2 Estimation of the coefficient of variation and mean positive partitions per well

To better understand how values of the CV change as a function of the level of concentration, we sought to estimate the true values of the CV by using a relatively large number of replicates (*n* = 46). Although the CV of the target concentration is widely used as an imprecision goal when determining the ddPCR aLLoQ, there is no consensus on the number of replicates, with 5–20 replicates being used (Bogožalec Košir et al., 2019; Dobnik et al., 2015; Milosevic et al., 2018). In the absence of agreement on the number of replicates, we used many replicates in combination with cumulative moving plots to assess our estimation of the mean positive partitions per well and the concentration CV (Figs. S2 and S3). What is of interest is if the cumulative moving parameter converges before the total number of replicates and remains stable (Fig. S3H). The cumulative moving mean positive partitions per well and cumulative moving concentration CV converged before the total number of replicates in all cases, indicating that the use of 46 replicates may provide useful estimates of the true values.

### 4.3 Analytical lower limit of quantification

We chose to set our aLLoQ to the level of concentration that would result in a coefficient of variation (CV) of 25% (Bogožalec Košir et al., 2019; Milosevic et al., 2018). While aLLoQs are often set using “less-than or equal to” a CV target (for example, ≤ 25%), the difficulty in finding the level of concentration that results in a specific CV can result in setting aLLoQs by satisfying the less than (<25%) rather than the equal to (= 25%) condition. However, this can cause variation in aLLoQs set using the same CV target; hypothetically, aLLoQs that have been set using levels of concentrations that resulted in a CV of 23% versus a CV of 13% are both valid for an aLLoQ based on ≤ 25%. The difficulty in repeatedly trying different levels of concentration to achieve a CV of 25% is a deterrent to attempts to satisfy the “equal to” condition. We attempted to develop a standardized approach by interpolating an aLLoQ using a regression fitted to observed CV and concentration values, referred to as a variance function or imprecision profile.

While variance functions for ddPCR assays have not yet been widely explored, various forms of variance functions have been used in diverse fields (Davidian and Carroll, 1987; Sadler, 2016). Variance functions can have many forms (Berweger et al., 1998). If the distribution of positive partitions per ddPCR well can be modeled by the Poisson distribution, the variance of the calculated copies per ddPCR well (see section 2.4.3) is dependent on the variance of the number of positive partitions per ddPCR well. For a Poisson distribution, the variance is equal to the expected value; thus, we expect the CV to monotonically scale with increasing number of positive partitions per ddPCR well because the expected value of the Poisson distribution is a scale parameter. With this in mind, we plotted the CV and mean copies per ddPCR well on a log_10_-log_10_ plot, observing a linear relationship between data points (Fig. 2). We also plotted this relationship using data published by others (Bogožalec Košir et al., 2019; Milosevic et al., 2018) and observed similar linear relationships despite varying numbers of replicates used to estimate CV values (Fig. 2). Overall, the imprecision profiles are similar. Interestingly, the imprecision profile for the HF183/BacR287 assay (multiplexed with an IAC) was similar to the hCYTB484 simplex assay (Fig. 2), which is supported by the relative absence of a quantification bias when duplexing on the ddPCR platform (Cao et al., 2015). Because of the agreement between linear regressions for our data and data published by others, we hypothesize that assays implemented on the Bio-Rad QX100/QX200 ddPCR platforms may share similar imprecision profiles. This is supported by the fact that ddPCR calculates a concentration from direct counts of many endpoint PCR results, eliminating some variability associated with individual assay performance. An interpolation approach using an imprecision profile to determining an aLLoQ can be helpful in allowing operators to set aLLoQs in a more standardized approach by providing a more precise estimation of the level of concentration that satisfies the aLLoQ definition rather than assaying various levels of concentration in attempts to satisfy the condition of less than or equal to target CV. The combination of relatively high values of *r*^2^ and low *p*-values (Fig. 2) leads us to believe that a linear regression fitted to log_10_ transformed CV values and log_10_ transformed copies per ddPCR well is a potential model for the imprecision profile of a variety of assays on the Bio-Rad QX100/QX200 ddPCR platforms for the range of copies per ddPCR well investigated in this study. Further research investigating more models, assays, and levels of concentration across different research groups performing ddPCR is warranted.

### 4.4 Comparing marker performance

We quantified the hCYTB484 marker on ddPCR at greater concentrations (copies/100 mL of wastewater) in both human feces and wastewater than previously-published TaqMan human mtDNA assays on qPCR (Caldwell et al., 2007; Schill and Mathes, 2008). However, we still quantified the hCYTB484 marker at lower concentrations than those of the HF183/BacR287 marker in wastewater (Fig. 4). In the freshwater samples, we observed the expected increasing levels of loading of the hCYTB484 and HF183/BacR287 markers with increasing drainage contribution from urban development (Fig. 5A and B). The two markers also had strong correlation with each other in freshwater samples (Spearman’s rank correlation coefficient of 0.85), with the HF183/BacR287 marker typically at higher levels of loadings (Fig. 5C).

ROC curves are a useful method of comparing detailed classifier performance because they communicate the dependency of sensitivity and specificity on threshold (Fawcett, 2006; Morrison et al., 2003). In some cases, different thresholds for declaring a sample as positive can result in dramatically different sensitivity and specificity performances being calculated (Layton et al., 2013). By showing how sensitivity and specificity vary with threshold, ROC curves communicate detailed performance characteristics crucial to selecting a threshold, facilitating standardization of the implementation of assays. In our ROC curves, we show that the hCYTB484 specificity dramatically increases when the threshold is moved from no amplification to the aLoD and has 100% specificity at the aLLoQ (Fig. 3). This communicates that although the hCYTB484 assay can exhibit low levels of false-positives, these false-positives can be largely avoided by using the aLoD or aLLoQ as the threshold, with the aLLoQ offering a very high specificity performance. Compared to the HF183/BacR287 marker, the hCYTB484 marker maintains higher sensitivity in human feces at all levels of threshold (Table 4). Through the ROC curve, we demonstrate the hCYTB484 marker to be widely distributed in individual human feces in concentrations above the aLLoQ, suggesting that the hCYTB484 marker is potentially reliable and useful in detecting human fecal sludges and other concentrated fecal waste streams where fewer individuals contribute to the waste. Such matrices are the most common form of fecal wastes globally where risks of exposure are highest (Berendes et al., 2018).

Because a marker with all of the ideal FST characteristics (Hagedorn et al., 2011) has proved to be elusive, “toolbox” approaches using multiple methods may be useful, recognizing that the strengths of one marker can complement the weaknesses of another marker (Boehm et al., 2013; Harwood et al., 2014). In this study, we developed a human mtDNA ddPCR assay, hCYTB484, and compared its performance characteristics with the HF183/BacR287 marker using ROC curves, showing how ROC curves can be used for detailed comparisons of assay performance. Considering the recommendations for validation studies of microbial FST markers in unproven settings before their use (Harris et al., 2016; Nshimyimana et al., 2017; Odagiri et al., 2015), aspects of mtDNA FST methods such as the direct targeting of host DNA and the wide distribution of mtDNA markers throughout diverse individuals illuminate mtDNA methods as a potentially useful set of tools in the FST toolbox.

### 4.5 Further considerations

Indeed, the use of human mtDNA as a fecal source tracking marker is a departure from the traditional paradigm of using microbial fecal indicators. As demonstrated by Zimmerman et al. (2014), human mtDNA was found in each laundry graywater sample at consistent levels of concentration while the human-associated *Bacteroides* marker used was not, despite the mean concentrations of both markers remaining similar. We note that the use of human mtDNA as an FST marker will require more studies to enable the interpretation of this marker. For example, because human mtDNA is found in cells not present in fecal matter, such assays can amplify human mtDNA from non-fecal origins. Human mtDNA marker copies in laundry greywater (Zimmerman et al., 2014) was orders of magnitude lower those found in wastewater (Caldwell et al., 2007; Schill and Mathes, 2008), suggesting that the level of concentration in the sample may serve as an indicator for non-fecal versus fecal contamination. Carry-over signals resulting from non-human animals consuming human fecal matter are also possible as human consumption of beef has resulted in beef mtDNA signal in human feces (Caldwell et al., 2007). How carry-over human mtDNA signal is influenced by ingestion of human fecal matter by non-human animals is a concern in areas where domestic animals are routinely exposed to environments contaminated with human feces (Harris et al., 2016) and in unsafe management of human feces disparately prevalent in low-income settings (Berendes et al., 2018). Human mtDNA markers have also been shown to persist in freshwater for relatively longer than HF183-based markers (He et al., 2015). These aspects of human mtDNA may make it a more “conservative” marker that correlates well with the presence of human activity and acts as a relatively persistent indicator of fecal pollution. Questions that are pertinent to the use of human mtDNA as an FST marker in environmental media need more studies, such as: typical concentrations in a variety of fecal and nonfecal samples, relative persistence in environmental media, cellular origins of fecal mtDNA, relationship with pathogen fate and transport in environmental media, and relationship with risk. Once human mtDNA as a marker of human fecal contamination has been better characterized, human mtDNA, as an unambiguous indicator of human influence, may complement other microbial-based FST markers that have been limited in their source-specificity (Mayer et al., 2018).

### 4.6 Limitations

Several limitations qualify the results of this study. Our specificity testing was limited to cows, pigs, chickens, and goats from the US to test specificity in this study. Although previous studies have shown human mtDNA to be highly specific to human feces (Caldwell et al., 2007; He et al., 2016; Schill and Mathes, 2008), further specificity testing including various species of relevance to other geographies (such as avian and non-cattle ruminant species) are useful for further assessment. While our aLoD experiments show agreement between theoretical calculations and observed ddPCR experiments, our use of the Poisson distribution to calculate our aLoD represents a theoretical aLoD that does not account for biases induced by the operator or equipment. Interpolation approaches to the determination of aLLoQ are helpful in that they may provide a standardized approach; however, because the imprecision profiles of ddPCR platforms have not been widely studied, the selection of a model for imprecision profile is not well guided by the literature. Our choice of a linear regression on log_10_ transformed values of both CV and the copies per ddPCR well was guided by the relationship between the expected value and variance for a Poisson distribution simulating the positive partitions per ddPCR well.

## 5 Conclusions

• We present a novel TaqMan chemistry assay for the ddPCR platform targeting human mtDNA, hCYTB484, and compared it with the HF183/BacR287 on ddPCR.
• We defined and established analytical limits for hCYTB484 and HF183/BacR287, presenting novel empirical evidence to validate the analytical limit of detection and a novel approach to the determination of analytical lower limit of quantification.
• The hCYTB484 assay was detected and quantifiable in 100% of human feces samples from the US, Mozambique, and Bangladesh, whereas HF183/BacR287 was detected in only 51%.
• The hCYTB484 displayed higher specificity performance in pig, chicken, and goat samples but lower specificity in cow samples.
• Using a receiver operating characteristic curve can provide valuable comparisons between assays and detailed analytical performance beyond scalar values of sensitivity and specificity.

## Declaration of competing interest

The authors declare that they have no known competing financial interests or personal relationships that could have appeared to influence the work reported in this paper.
