# Supplementary material for: A novel droplet digital PCR human mtDNA assay for fecal source tracking
Source: Water Res. 2020 Sep 15;183:116085. doi: 10.1016/j.watres.2020.116085 (PMC7495096; doi:10.1016/j.watres.2020.116085)
Supplement: Multimedia component 3 [file mmc3.docx]

**Supplementary Material for:**

**A Novel Droplet Digital PCR Human mtDNA Assay for Fecal Source Tracking**

*Kevin Zhu^a^, Brittany Suttner^a^, Amy Pickering ^b^, Konstantinos T. Konstantinidis^a^, Joe Brown^a#^*

*^a^ School of Civil and Environmental Engineering, Georgia Institute of Technology, Atlanta Georgia, United States of America*

*^b^ Civil and Environmental Engineering, Tufts University, Medford, Massachusetts, United States of America*

^#^ corresponding author email: [joe.brown@ce.gatech.edu](mailto:joe.brown@ce.gatech.edu)

# Materials and Methods

**Assay design**

While implementing previously-designed human mtDNA assays (Caldwell et al., 2007; Schill and Mathes, 2008) on the ddPCR platform, we observed notable noise when differentiating between positive and negative partitions. One assay was designed as a mismatch amplification mutation assay by intentionally designing penultimate primer mismatches to increase species-specificity (Caldwell et al., 2007), potentially inducing PCR inefficiencies and leading to increased noise on ddPCR (Stadhouders et al., 2010). Another assay (Schill and Mathes, 2008) contains a probe that spans several population-variable polymorphisms (Ablimit et al., 2013; Hwa et al., 2010; Lee et al., 2002). Because the mitochondrial genome is maternally inherited, polymorphisms have been studied for their utility in identifying human individuals from different matrilineages. Several studies have investigated the use of polymorphisms within the cytochrome *b* gene to discriminate between populations of people, finding that combinations of polymorphisms can be used to discriminate between different populations of people (Ablimit et al., 2013; Hwa et al., 2010). The discriminatory power of these polymorphisms alludes to the potential for geographic variations in performance of mtDNA-based methods because mismatches between template and oligonucleotides can result in inefficiencies in PCR (Letowski et al., 2004; Stadhouders et al., 2010).

**Human feces samples:** From the US, we obtained healthy, pre-challenge feces samples from 11 adults (18 to 50 years of age) enrolled in a norovirus challenge study (trial registration number: NCT00674336). From Mozambique, we obtained pre-intervention feces samples from 71 children under 4 years of age enrolled in an urban sanitation intervention study (trial registration number: NCT02362932). From Bangladesh, we obtained feces samples from 142 children under 5 years of age enrolled in a water treatment intervention study (trial registration number: NCT02606981). All feces samples were stored at -80°C prior to nucleic acid extraction.

**Non-human feces samples:** To test specificity against non-human sources, we collected fecal samples from 22 cows, 35 pigs, and 22 goats from farms located in north Georgia using sterile 15 mL tubes. We transported cow and pig samples in a 1:1 mixture with Cary-Blair media due to the travel duration to the farms and because the Cary-Blair transport medium was developed as a low nutrient transport medium for the preservation of gram negative and anaerobic organisms in fecal specimens (Cary and Blair, 1964). We transported the goat samples from collection on ice because it was a shorter duration, processing samples within 2 hours of collection. We collected chicken litter from 8 chicken houses by outlining a 10 cm by 10 cm area along the drinking lines, homogenizing the litter contained within the grid and collecting approximately 50 mL of the litter into a sterile bag. We collected samples from 3 different locations within each house to create composite samples for each chicken house. We transported chicken litter samples on ice.

**Wastewater and freshwater samples:** We collected approximately 1 L of each wastewater sample and transported wastewater samples on ice, filtering the samples within 6 hours of collection. We collected the freshwater samples by lowering a sterile 2 L glass bottle from a bridge over the river, rinsing the collection bottle twice with river water before collecting the sample. We transported wastewater and freshwater samples on ice, filtering the samples within 6 hours of collection. For the freshwater samples, we used the sketa22 assay to as a sample processing control (Haugland et al., 2010).

**Nucleic acid extractions**

For all human feces samples, we started extractions by gently mixing the feces sample with a sterile inoculating loop and extracting from approximately 0.1 grams of feces sample.

**End-point PCR amplifications**

To test for cross-reactivity of our mtDNA marker against non-human mtDNA, we tested each cow and pig sample for the presence of the respective host-animal mtDNA (Caldwell et al., 2007) using end-point PCR. All cow and pig samples were confirmed positive for their respective host species mtDNA. We also used end-point PCR to generate PCR product for cloning prior to sequencing in the verification of positive standards and for confirmation of target sequences. Lastly, for each assay, we checked for the presence of primer dimers by visualizing end-point PCR products on a fluorescent gel; we did not detect presence of primer dimers for both assays. For each 20 μL end-point PCR reaction, we used 10 μL TaKaRa (KUStsu, Shiga, Japan) Premix Ex Taq™ 2x concentration, with 300 nM of each primer, and 2 μL of template. We used a thermocycling routine consisting of 98°C for 10 seconds, 55°C for 30 seconds, 72°C for 60 seconds, and 72°C for 7 minutes. For visualization, we used 1% GelRed stain from Biotium (Fremont, CA, US) with a 2.5% agarose gel and a 20 bp molecular ruler from Bio-Rad Laboratories, Inc. (Hercules, CA, US).

**Droplet digital PCR experiments**

We ordered all primers (standard desalting purification) used in this study (Table 1) as custom-manufactured DNA oligos from IDT (Coralville, IA, US). We obtained the hCYTB484 probe as a custom-manufactured probe (HPLC purification) with Internal ZEN™ Quencher Placement from IDT. We ordered the BacP234MGB and BacP234IAC probes from Applied Biosystems™ (Waltham, MA, US) as TaqMan® minor groove binder (MGB) probes (HPLC purification).

For each assay, we conducted a series of experiments to optimize the annealing temperature. First, we ran a temperature gradient spanning approximately 8°C; then, we ran a finer scale temperature gradient (spanning approximately 2°C) by identifying the highest temperatures in which the separation between negative and positive bands reached a limit in the previous gradient. We selected an annealing temperature from the finer scale gradient that gave us the most separation while remaining a relatively high temperature to avoid non-specific amplification. We also experimented with 94°C, 95°C, and 96°C denaturation cycles, finding that 95°C provided the best separation between positive and negative partition signals for the assays used in this study.

**Intra-stool marker variability**

To assess sampling variability within the human fecal samples, we extracted a subset (7%) of the human feces samples as biological duplicates (duplicate DNA extracts of the same fecal sample). For the hCYTB484 assay, we calculated a mean percent difference between biological duplicates of 24% (standard deviation of 27%) and observed no changes in detection status (all biological replicates were quantifiable). For the HF183/BacR287 assay, we observed two cases of changes in detection status between biological replicates.

**Sensitivity and specificity testing**

We assayed each cow, pig, chicken, and goat sample with HF183/BacR287 and hCYTB484 on ddPCR with 25% technical replicates. Any non-human sample that returned above our aLoD (3 positive partitions), we ran endpoint PCR and gel electrophoresis to confirm the size of the PCR product. We assayed each human feces sample using hCYTB484 and HF183/BacR287 on ddPCR with 25% technical replicates.

**Tables**

**Table S1.** Results of analyzing no-template controls (UV-treated molecular-grade water) on ddPCR for both assays.

| **Number of Positive Partitions in a Well** | **Frequency of Occurrence** | | **Relative to aLoD** |
| --- | --- | --- | --- |
|  | **HF183/BacR287**  (n = 94) | **hCYTB484**  (n = 93) |  |
| 0 | 97.85 % | 95.74 % | Below aLoD |
| 1 | 2.15 % | 3.19 % |  |
| 2 | 0.00 % | 0.00 % |  |
| 3 | 0.00 % | 0.00 % | Above aLoD |
| 4 | 0.00 % | 1.06 % |  |

**Figures**


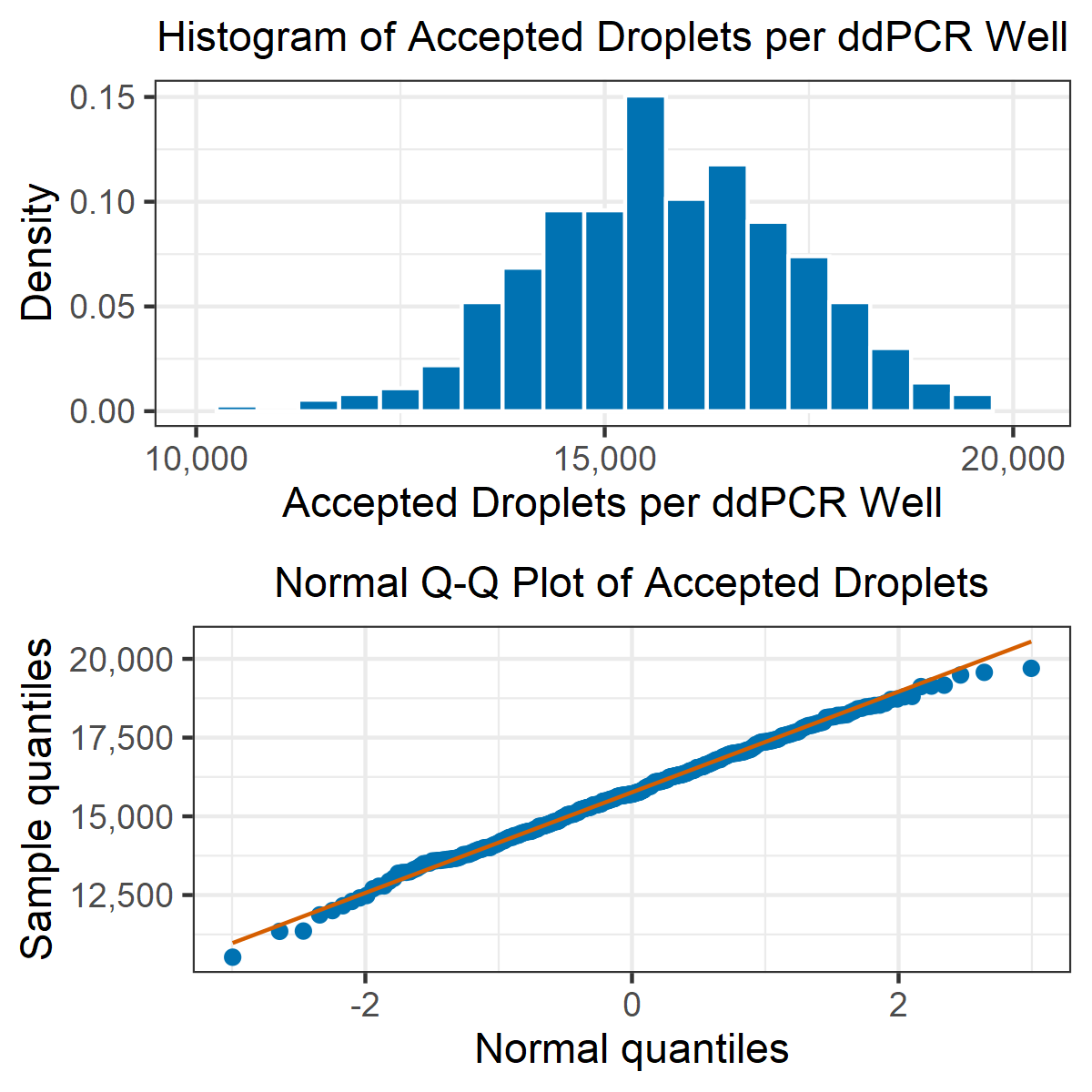


**Figure S1.** Histogram of the accepted partitions (top) and quantile-quantile plot comparing the distribution of accepted partitions with a theoretical normal distribution along with a quantile-quantile line for comparison (bottom). These plots show the numbers of accepted partitions for the ddPCR experiments conducted in this study on the Bio-Rad QX200 platform.


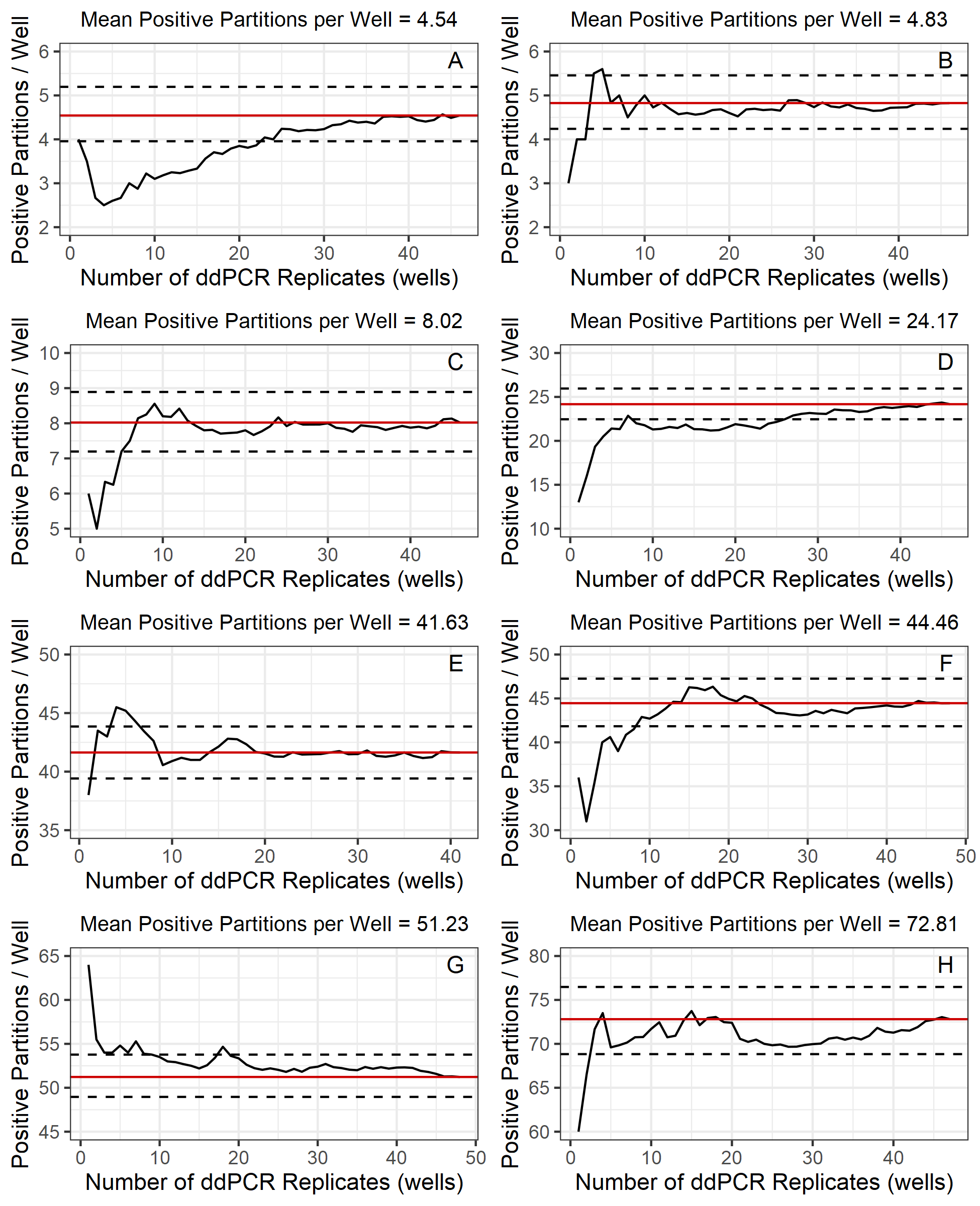


**Figure S2.** Cumulative moving plots of mean positive partitions per well for a range of mean positive partitions per well. The mean positive partitions per well calculated from total number of replicates is shown as the red solid horizontal line and bootstrapped 95% confidence interval is shown as the black dotted horizontal lines.


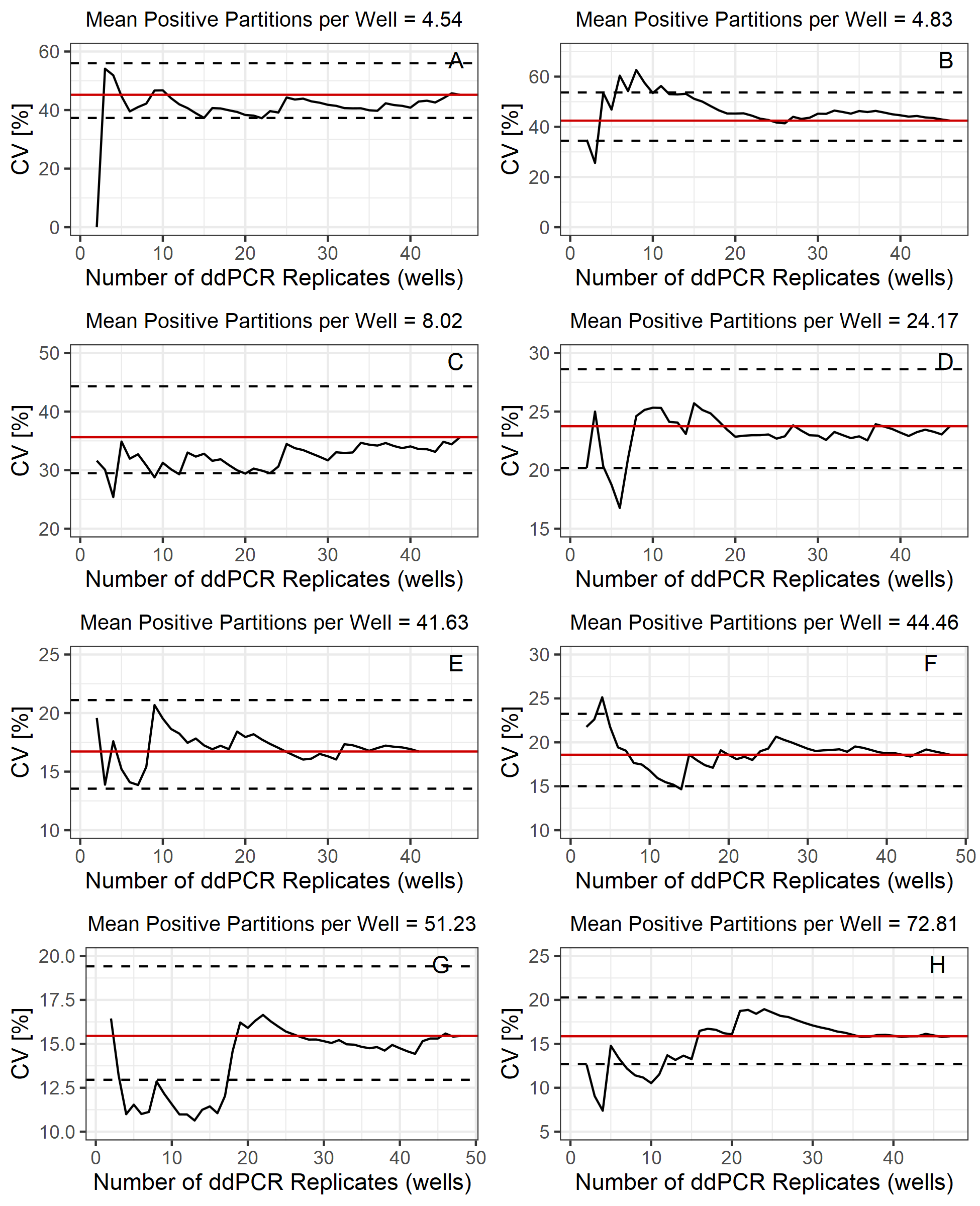


**Figure S3.** Cumulative moving plots of CV for a range of mean positive partitions per well. The CV calculated from total number of replicates is shown as the red solid horizontal line and bootstrapped 95% confidence interval is shown as the black dotted horizontal lines.


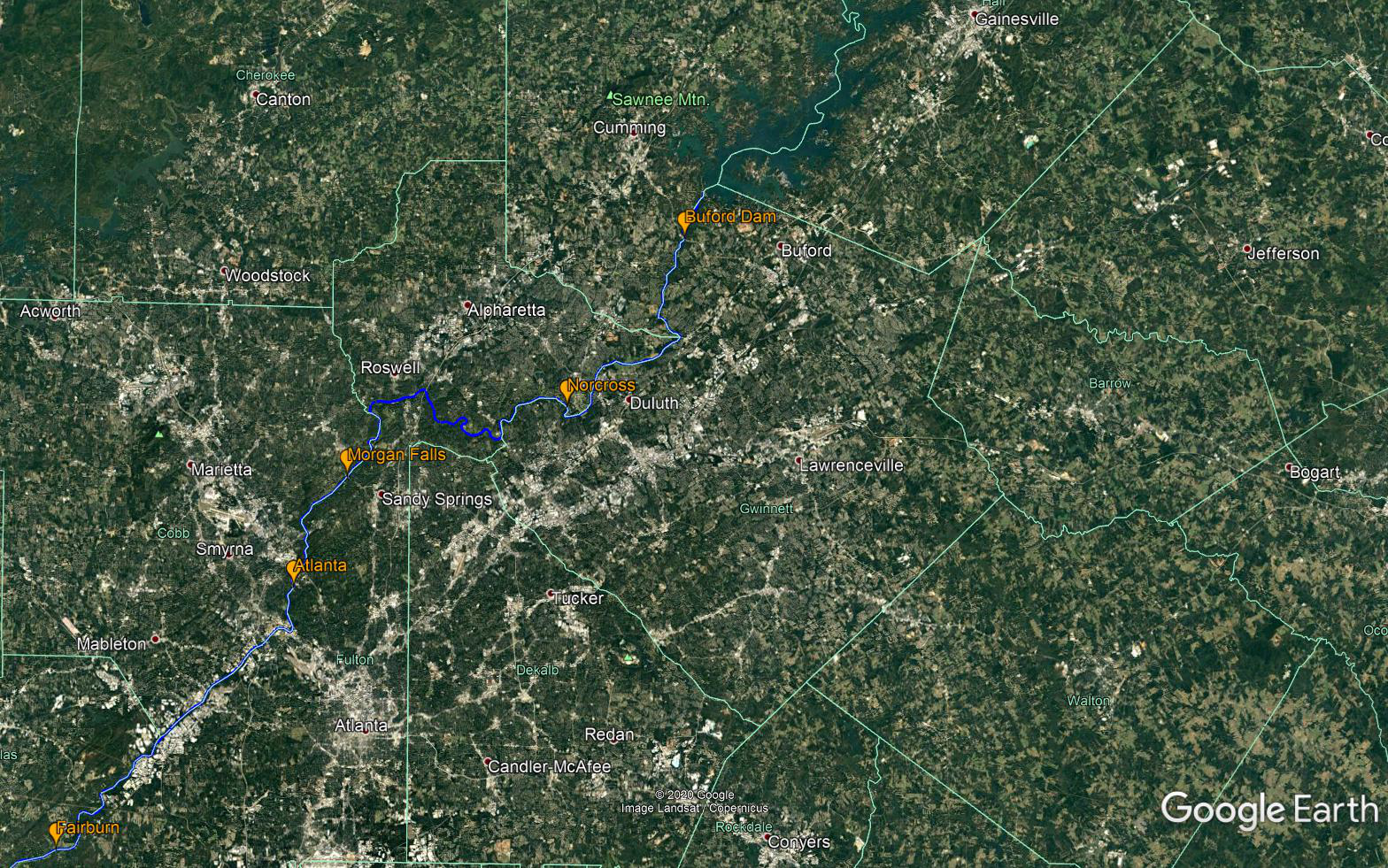


**Figure S4.** Sampling locations along the Chattahoochee River shown as orange location pins along the river (in blue). The first sampling location is approximately 5 km downstream of the outlet of Buford Dam, which dams Lake Lanier (the large reservoir in the top right hand corner of the map).

**Poisson Maximum Likelihood Estimation**

Suppose X_1_,…,X_n_ are random samples from a Poisson distribution with parameter **λ**. To derive the maximum likelihood estimator (in brief), we start with the Poisson probability mass function:

$$p\left( x \right)=\frac{\lambda^{X}e^{-\lambda}}{x!}, x=0,1,2,\ldots, \lambda>0$$

Likelihood function:

$$L\left( \lambda\right)=\prod_{i=1}^{n} \frac{\lambda^{x_{i}}e^{-\lambda}}{x_{i}!}=\frac{\lambda^{\sum_{i=1}^{n} x_{i}}e^{-n\lambda}}{\prod_{i=1}^{n} x_{i}!}$$

Taking the natural logarithm:

$$\ln L\left( \lambda\right)=\sum_{i=1}^{n} x_{i}\ln\lambda-n\lambda-\sum_{i=1}^{n} \ln\left( x_{i}! \right)$$

Differentiating with respect to λ:

$$\frac{d \ln L\left( \lambda\right)}{d\lambda}=\frac{\sum_{i=1}^{n} x_{i}}{\lambda}-n$$

Setting the derivative (right hand side) equal to zero:

$$\frac{\sum_{i=1}^{n} x_{i}}{\lambda}-n=0$$

$$\lambda=\frac{\sum_{i=1}^{n} x_{i}}{n}=\bar{x}$$

Therefore, the MLE of λ is the sample mean:

$$\hat{\lambda}=\bar{x}$$

**References**

Ablimit, A., Qin, W., Shan, W., Wu, W., Ling, F., Ling, K.H., Zhao, C., Zhang, F., Ma, Z., Zheng, X., 2013. Genetic diversities of cytochrome B in Xinjiang Uyghur unveiled its origin and migration history. BMC Genet. 14, 1. https://doi.org/10.1186/1471-2156-14-100

Caldwell, J.M., Raley, M.E., Levine, J.F., 2007. Mitochondrial Multiplex Real-Time PCR as a Source Tracking Method in Fecal-Contaminated Effluents. Environ. Sci. Technol. 41, 3277–3283. https://doi.org/10.1021/es062912s

Cary, S.G., Blair, E.B., 1964. New Transport Medium for Shipment of Clinical Specimens. I. Fecal Specimens. J. Bacteriol. 88, 96–98.

Haugland, R.A., Varma, M., Sivaganesan, M., Kelty, C., Peed, L., Shanks, O.C., 2010. Evaluation of genetic markers from the 16S rRNA gene V2 region for use in quantitative detection of selected Bacteroidales species and human fecal waste by qPCR. Syst. Appl. Microbiol. 33, 348–357. https://doi.org/10.1016/j.syapm.2010.06.001

Hwa, H.L., Ko, T.M., Chen, Y.C., Chang, Y.Y., Tseng, L.H., Su, Y.N., Lee, J.C.I., 2010. Study of the cytochrome b gene sequence in populations of Taiwan. J. Forensic Sci. 55, 167–170. https://doi.org/10.1111/j.1556-4029.2009.01195.x

Lee, S.D., Lee, Y.S., Lee, J. Bin, 2002. Polymorphism in the mitochondrial cytochrome B gene in Koreans. Int. J. Legal Med. 116, 74–78. https://doi.org/10.1007/s004140100238

Letowski, J., Brousseau, R., Masson, L., 2004. Designing better probes: effect of probe size, mismatch position and number on hybridization in DNA oligonucleotide microarrays. J. Microbiol. Methods 57, 269–278. https://doi.org/10.1016/j.mimet.2004.02.002

Schill, W.B., Mathes, M. V., 2008. Real-time PCR detection and quantification of nine potential sources of fecal contamination by analysis of mitochondrial Cytochrome b targets. Environ. Sci. Technol. 42, 5229–5234. https://doi.org/10.1021/es800051z

Stadhouders, R., Pas, S.D., Anber, J., Voermans, J., Mes, T.H.M., Schutten, M., 2010. The Effect of Primer-Template Mismatches on the Detection and Quantification of Nucleic Acids Using the 5′ Nuclease Assay. J. Mol. Diagnostics 12, 109–117. https://doi.org/10.2353/jmoldx.2010.090035
